# Supplementary material for: Green, Brown, and Gray: Associations between Different Measurements of Land Patterns and Depression among Nursing Students in El Paso, Texas
Source: Int J Environ Res Public Health. 2020 Nov 4;17(21):8146. doi: 10.3390/ijerph17218146 (PMC7662514; doi:10.3390/ijerph17218146)
Supplement: Supplementary file 1 [file ijerph-17-08146-s001.pdf]

Supplementary Material

Land patterns examples

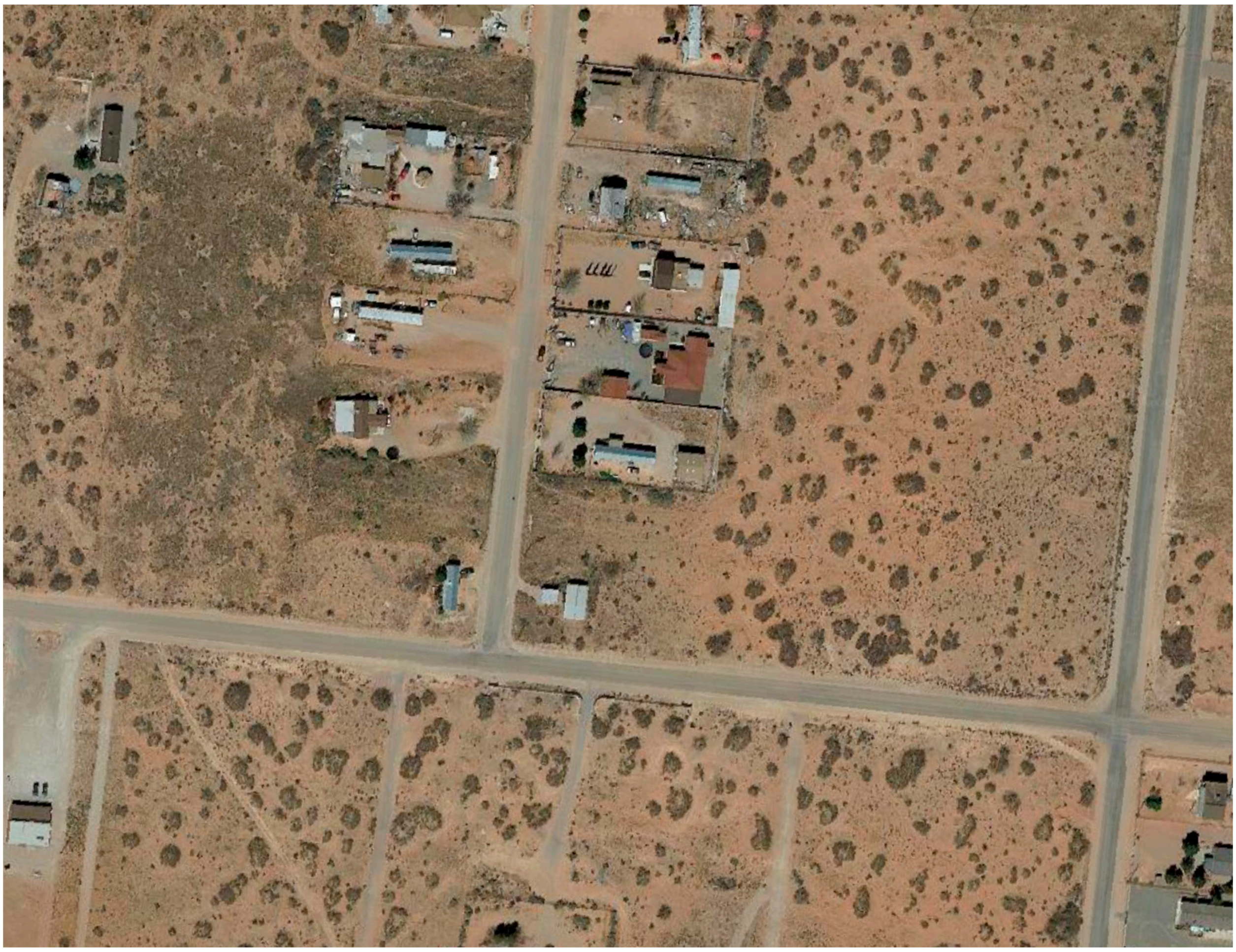

Figure S1. Example of relatively high brown, moderate green, low grey area

.

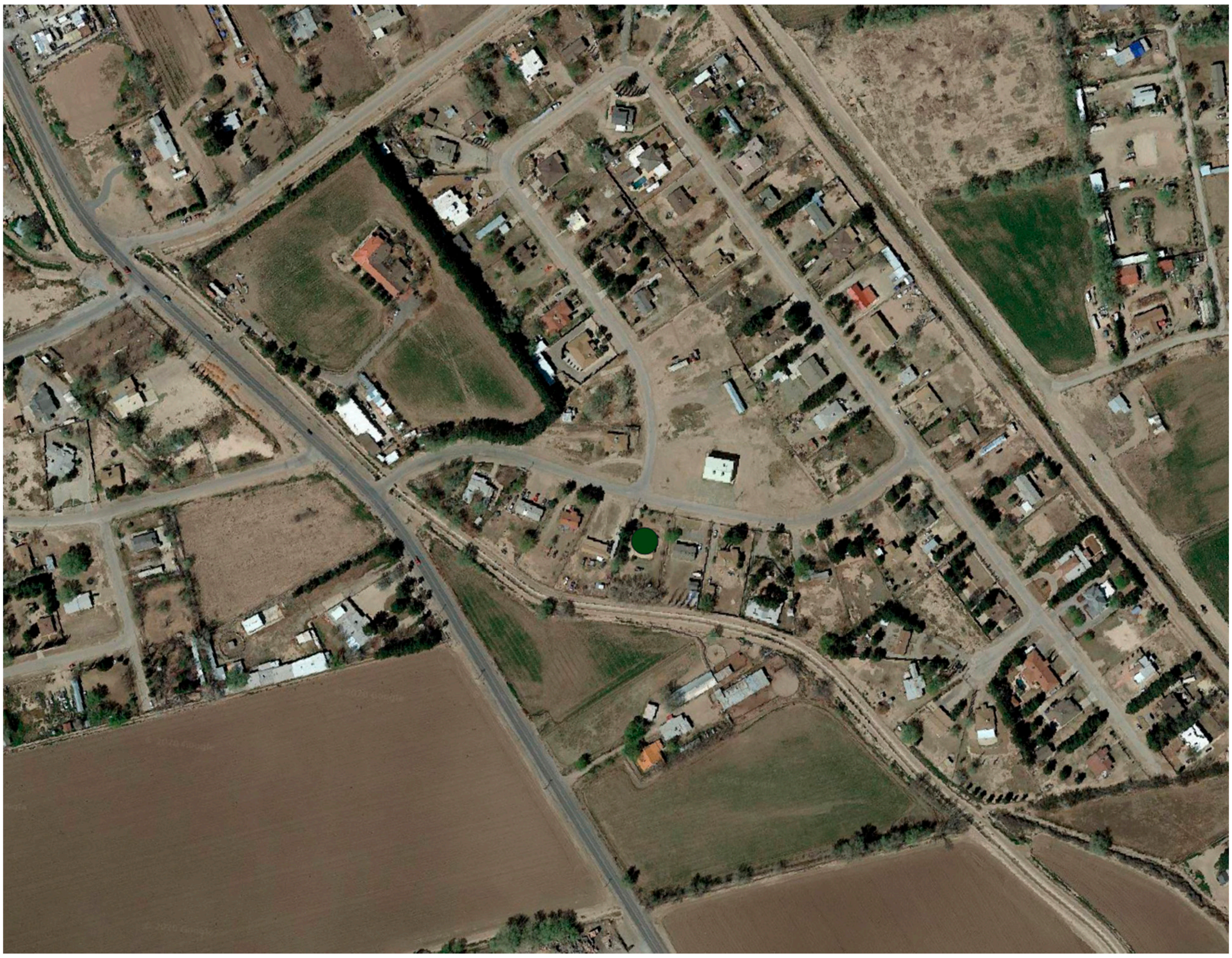

Figure S2. Example of relatively high brown, high green, and low grey area.

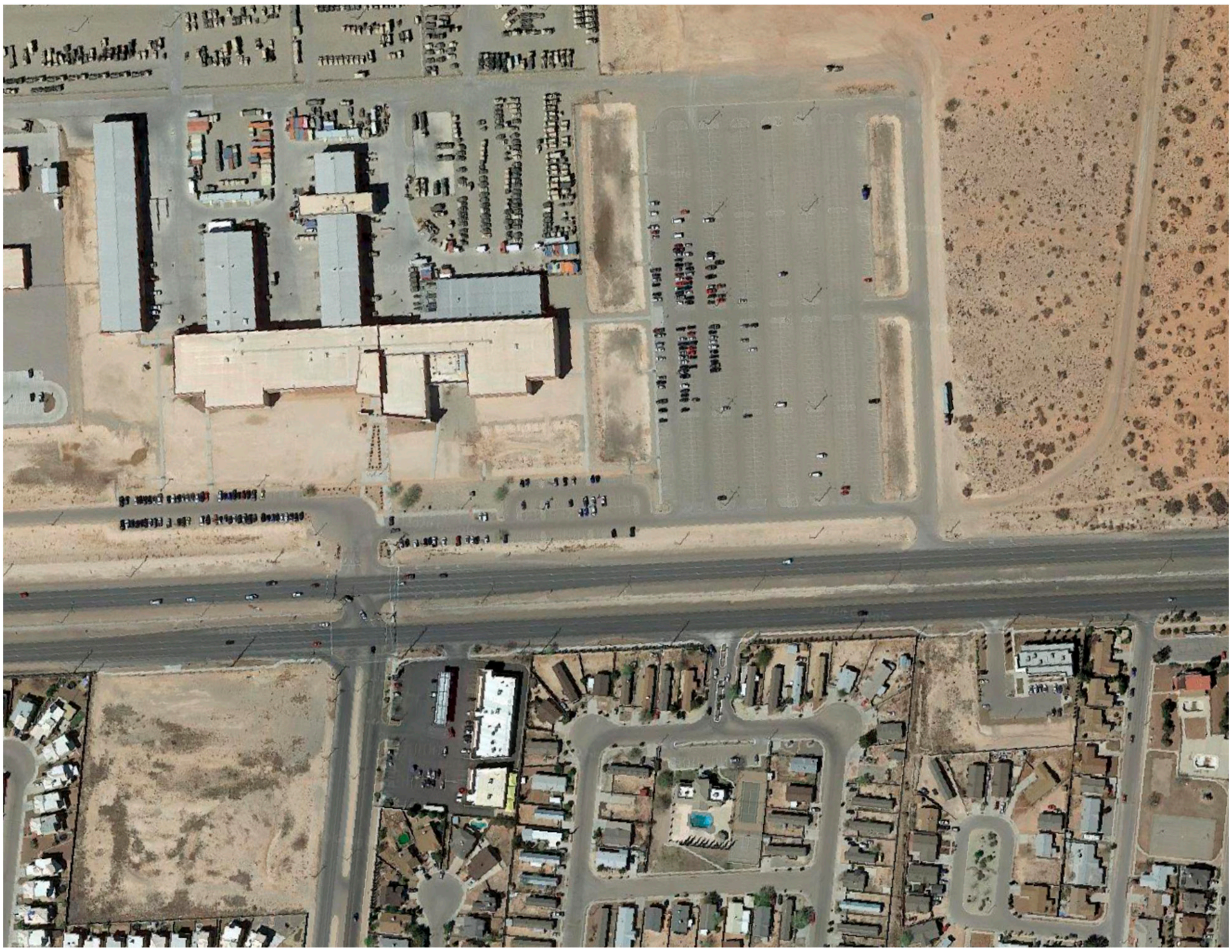

Figure S3. Example of relatively moderate brown, low green, and moderate grey area.

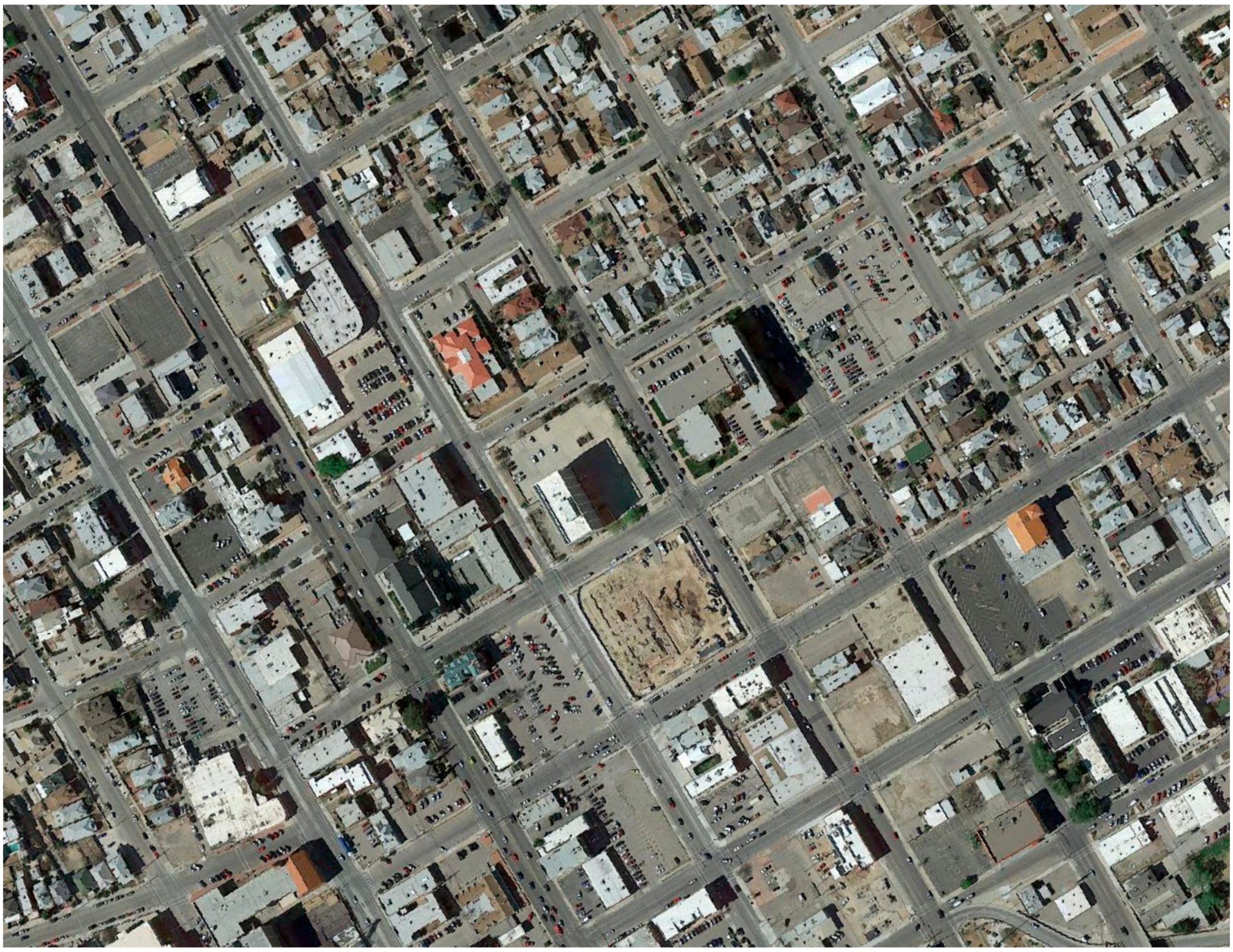

Figure S4. Example of relatively low brown, low green, and high grey area.

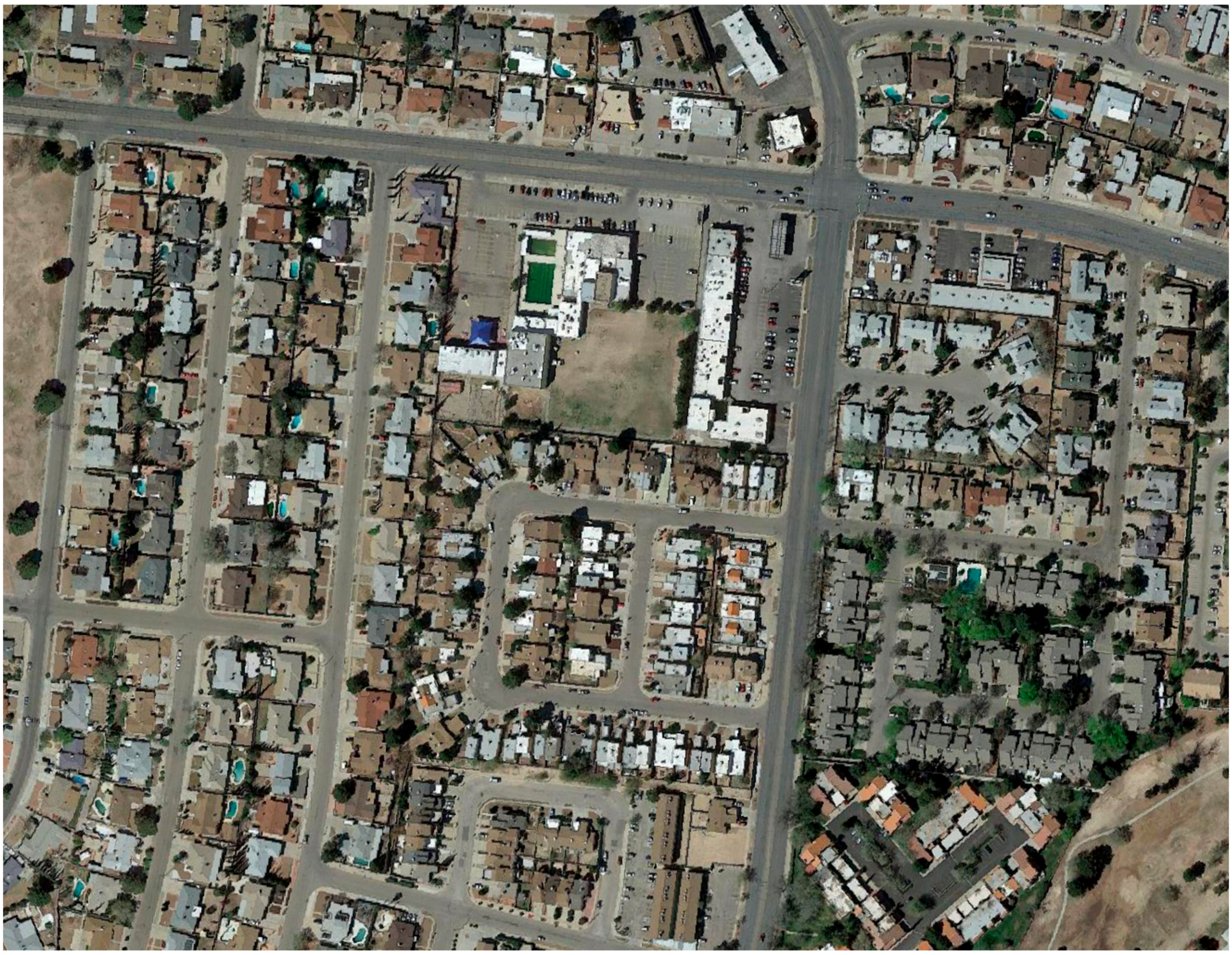

Figure S5. Example of relatively low brown, moderate green, and high grey area.

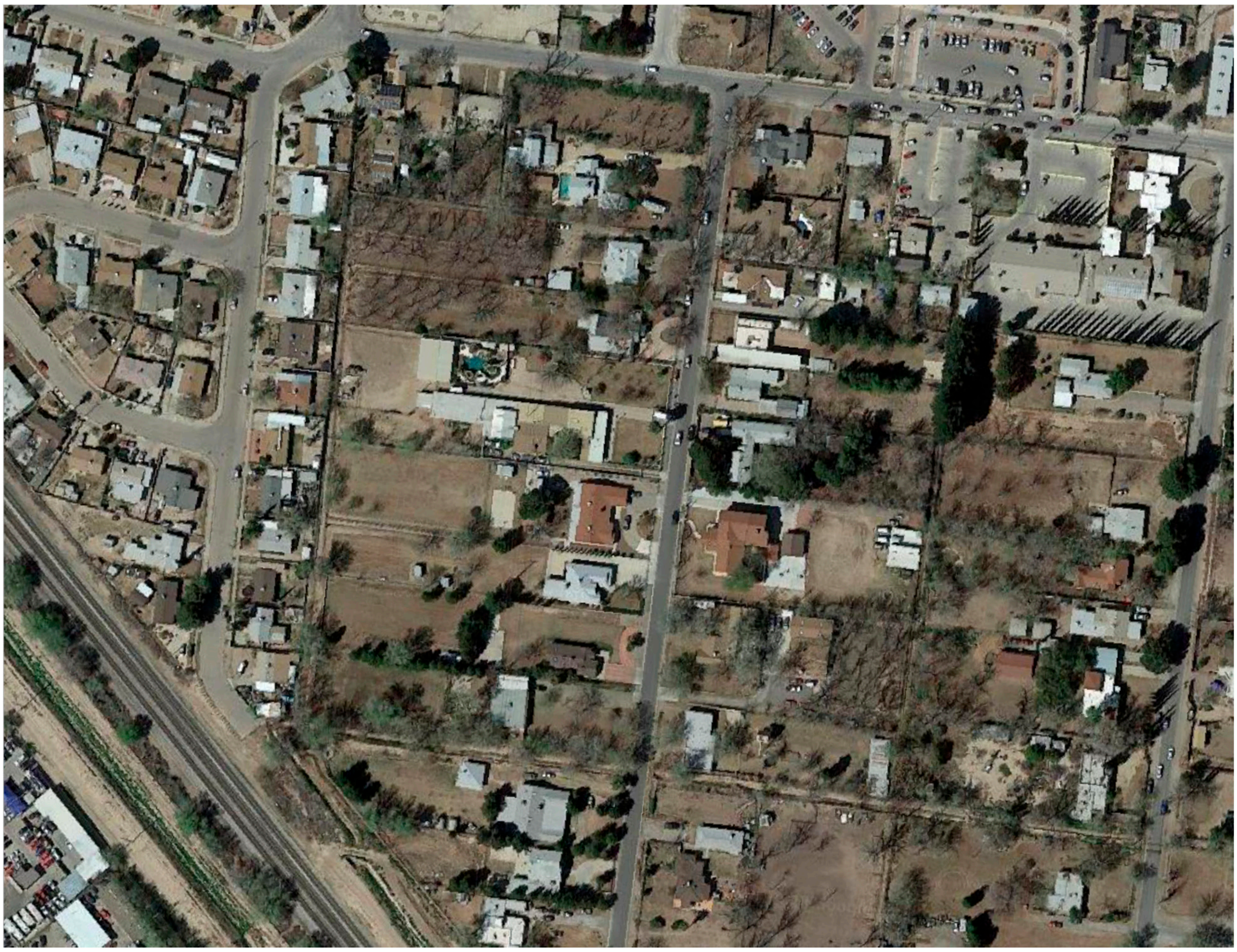

Figure S6. Example of relatively moderate brown, high green, and moderate grey area.

File S1

1. Questions to build the social support index

1. Is there someone available to you whom you can count on to listen to you when you need to talk?
2. Is there someone available to give you good advice about a problem?
3. Is there someone available to you who shows you love and affection?
4. Is there someone available to help you with daily chores?
5. Can you count on anyone to provide you with emotional support (talking over problems or helping you make a difficult decision)?
6. Do you have as much contact as you would like with someone you feel close to, someone in whom you can trust and confide?
7. Are you currently married or living with a partner?

With the exception of question 7 in which the possible answer were 1= "yes" or 2="no," the other questions had the following five alternatives: 1= "None of the time," 2= "A little of the time," 3= "Some of the time," 4= "Most of the time," and 5= "All the time."

2. Questions to build the peer attachment index

1. I like to get my friend's point of view on things I'm concerned about.
2. My friends can tell when I'm upset about something.
3. When we discuss things, my friends care about my point of view.

4. Talking over my problems with friends makes me feel ashamed or foolish.
5. I wish I had different friends.
6. My friends understand me.
7. My friends encourage me to talk about my difficulties.
8. My friends accept me as I am.
9. I feel the need to be in touch with my friends more often.
10. My friends don't understand what I'm going through these days.
11. I feel alone or apart when I am with my friends.
12. My friends listen to what I have to say.
13. I feel my friends are good friends.
14. My friends are fairly easy to talk to.
15. When I am angry about something, my friends try to be understanding.
16. My friends help me to understand myself better.
17. My friends care about how I am feeling.
18. I feel angry with my friends.
19. I can count on my friends when I need to get something off my chest.
20. I trust my friends.
21. My friends respect my feelings.
22. I get upset a lot more than my friends know about.
23. It seems as if my friends are irritated with me for no reason.
24. I can tell my friends about my problems and troubles.
25. If my friends know something is bothering me, they ask me about it.

Questions could be answered with 1= "Almost Never or Never True," 2= "Not Very Often True," 3= "Sometimes True," 4= "Often True," 5= "Almost Always or Always True."

3. Questions to build the physical−activity index

DURING THE PAST YEAR, what was your average time PER WEEK spent at each of the following physical activities?

1. Standing or walking around at work or at home
2. Walking for exercise or walking for transportation or errands
3. Running or jogging
4. Bicycling (include stationary machine)
5. Ball games (basketball, football, tennis, etc.)
6. Other aerobic exercise (aerobic dance, ski machine)
7. Lap swimming
8. Other vigorous activities (e.g., mowing lawn, climbing stairs)
9. Weight training or resistance exercises
10. Meditation or yoga

Questions could be answered with 0= "0 minute," 1= "1−5 minutes," 2= "6−20 minutes," 3= "21−59 minutes," 4= "1−2 hours," 5= "3−6 hours," 6= "7−10 hours," 7= "11 hours or more."

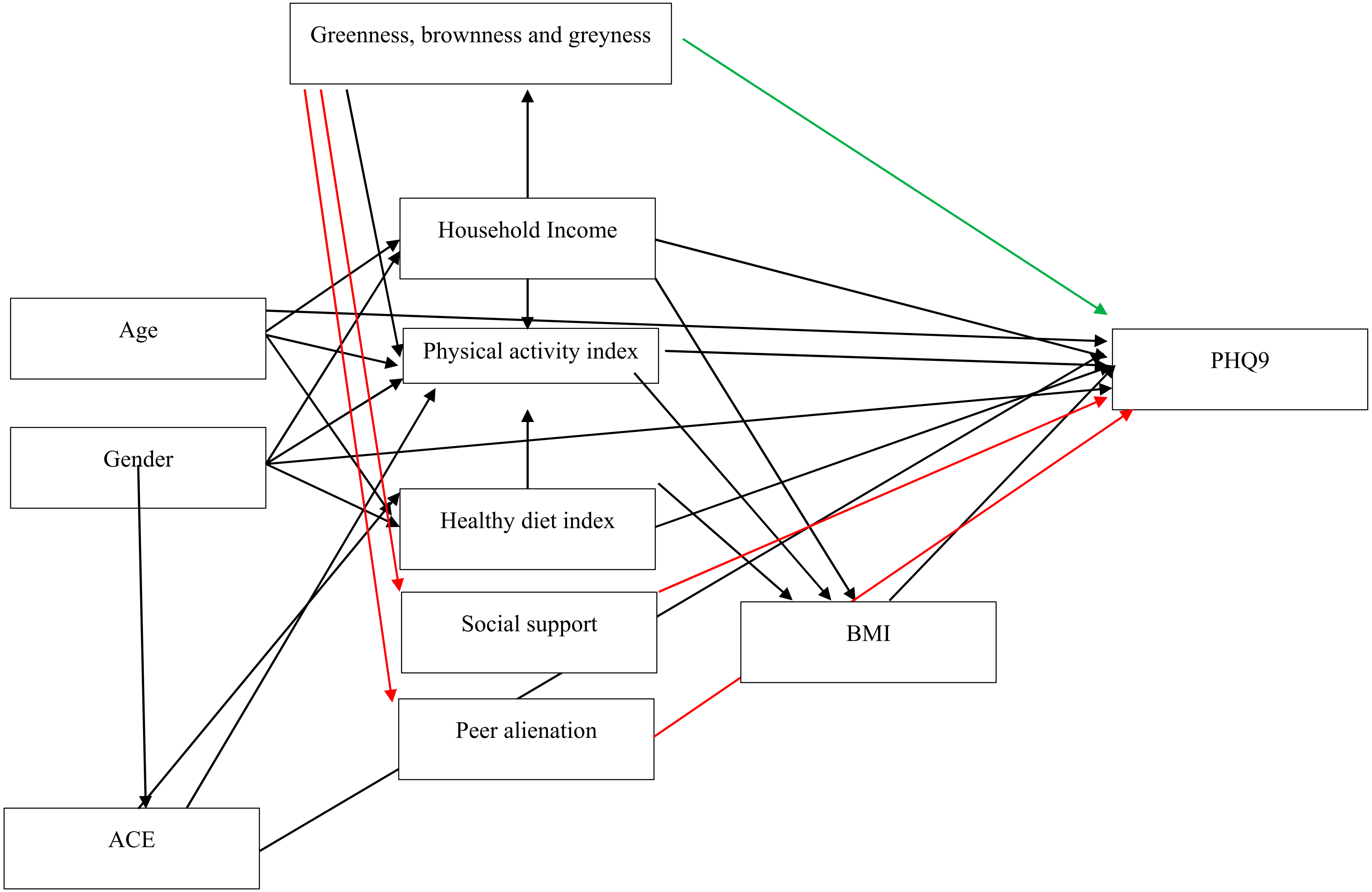
Greenness, brownness and greyness
Household Income
Age
Physical activity index
PHQ9
Gender
Healthy diet index
Social support
BMI
Peer alienation
ACE

Figure S7. Structural equation model diagram to estimate direct and indirect associations between PHQ9 and 'brownness and greenness' controlling for nine covariates. (This model suggests in the green arrow a direct association which guides the presentation of the results throughout the manuscript between greenness, brownness, or greyness with depression. Red arrows capture the association between greenness, brownness or greyness over the outcome of interest, mediated by social support and peer alienation. Black arrows are both direct and indirect associations between covariates and the outcome of interest. This figure is used to present results in the manuscript.

Table S1. Direct effects to predict depression with greenness at 250 m with four distributions (Poisson, Negative binomial, Gamma and Normal (log of depression)) (*n* = 393).

	**Poisson Distribution**			**Negative Binomial Distribution**			**Gamma Distribution**			**Normal Distribution**		
	Coefficient	95%	CI	Coefficient	95%	CI	Coefficient	95%	CI	Coefficient	95%	CI
Adverse Childhood Experiences	0.094	0.076	0.113	0.086	0.053	0.119	0.082	0.047	0.118	0.081	0.041	0.121
Social support index	−0.021	−0.029	−0.013	−0.024	−0.039	−0.009	−0.025	−0.041	−0.008	−0.034	−0.052	−0.017
Attachment to peers index	0.038	0.029	0.047	0.040	0.024	0.056	0.040	0.022	0.057	0.036	0.018	0.055
Healthy−food index	−0.022	−0.076	0.032	−0.025	−0.120	0.069	−0.025	−0.123	0.074	−0.020	−0.125	0.086
Physical−index activity	−0.087	−0.128	−0.045	−0.069	−0.139	0.000	−0.066	−0.136	0.004	−0.098	−0.177	−0.019
Body mass index	0.008	0.001	0.015	0.009	−0.004	0.023	0.009	−0.005	0.024	0.013	−0.002	0.027
Household income	−0.009	−0.016	−0.002	−0.009	−0.021	0.003	−0.009	−0.021	0.003	−0.011	−0.044	0.022
Age	0.258	0.150	0.365	0.279	0.103	0.456	0.288	0.112	0.464	−0.015	−0.029	−0.002
Gender	−0.006	−0.023	0.012	−0.010	−0.040	0.019	−0.012	−0.042	0.018	0.296	0.102	0.490
Greenness at 250 m	−0.606	−1.501	0.289	−0.647	−2.078	0.785	−0.719	−2.135	0.696	−0.671	−2.083	0.741
AIC	2464.975			2144.323			2118.956			942.4709		
BIC	2508.687			2192.008			2166.641			990.1566		

Table S2. Direct effects to predict depression with greenness at 500 m with four distributions (Poisson, Negative binomial, Gamma and Normal (log of depression)) (n = 393).

	**Poisson Distribution**			**Negative Binomial Distribution**			**Gamma Distribution**			**Normal Distribution**		
	Coefficient	95%	CI	Coefficient	95%	CI	Coefficient	95%	CI	Coefficient	95%	CI
Adverse Childhood Experiences	0.094	0.075	0.112	0.085	0.052	0.119	0.082	0.047	0.118	0.080	0.040	0.120
Social support index	−0.022	−0.030	−0.014	−0.024	−0.039	−0.009	−0.025	−0.041	−0.008	−0.034	−0.052	−0.017
Attachment to peers index	0.038	0.029	0.047	0.040	0.024	0.056	0.040	0.022	0.057	0.037	0.018	0.055
Healthy−food index	−0.023	−0.077	0.031	−0.026	−0.121	0.070	−0.024	−0.123	0.075	−0.019	−0.125	0.087
Physical−index activity	−0.087	−0.128	−0.045	−0.069	−0.139	0.001	−0.066	−0.136	0.004	−0.097	−0.176	−0.017
Body mass index	0.008	0.000	0.015	0.009	−0.004	0.023	0.009	−0.005	0.024	0.013	−0.002	0.028
Household income	−0.007	−0.024	0.011	−0.011	−0.041	0.019	−0.013	−0.043	0.018	−0.011	−0.045	0.023
Age	−0.009	−0.016	−0.001	−0.009	−0.021	0.003	−0.009	−0.021	0.003	−0.015	−0.029	−0.002
Gender	0.258	0.151	0.365	0.281	0.105	0.458	0.289	0.113	0.466	0.298	0.104	0.492
Greenness at 500 m	−0.280	−1.570	1.009	−0.559	−2.035	0.917	−0.574	−2.088	0.940	−0.629	−2.086	0.829
AIC	2454.407			2135.191			2109.857			939.8363		
BIC	2498.035			2182.815			2157.482			987.4608		

Table S3. Direct effects to predict depression with greenness at 1000 m with four distributions (Poisson, Negative binomial, Gamma and Normal (log of depression)) (n = 393).

	**Poisson**			**Negative Binomial**			**Gamma**			**Normal**		
	Coefficient	95%	CI	Coefficient	95%	CI	Coefficient	95%	CI	Coefficient	95%	CI
Adverse Childhood Experiences	0.094	0.076	0.113	0.086	0.052	0.119	0.082	0.047	0.118	0.081	0.041	0.121
Social support index	−0.021	−0.029	−0.013	−0.024	−0.039	−0.009	−0.025	−0.041	−0.008	−0.034	−0.052	−0.017
Attachment to peers index	0.039	0.030	0.048	0.040	0.024	0.056	0.040	0.023	0.057	0.036	0.018	0.055
Healthy−food index	−0.022	−0.076	0.032	−0.025	−0.120	0.070	−0.024	−0.122	0.075	−0.019	−0.125	0.086
Physical−index activity	−0.087	−0.128	−0.045	−0.069	−0.138	0.001	−0.065	−0.135	0.004	−0.097	−0.177	−0.018
Body mass index	0.008	0.000	0.015	0.009	−0.004	0.022	0.009	−0.005	0.023	0.012	−0.003	0.027
Household income	−0.009	−0.016	−0.002	−0.009	−0.021	0.003	−0.009	−0.021	0.003	−0.015	−0.029	−0.002
Age	0.258	0.150	0.365	0.280	0.104	0.456	0.288	0.112	0.464	0.298	0.104	0.492
Gender	−0.006	−0.024	0.011	−0.010	−0.040	0.019	−0.012	−0.042	0.018	−0.011	−0.044	0.022
Greenness at 1000 m	−0.570	−1.532	0.392	−0.527	−2.054	1.000	−0.522	−2.100	1.056	−0.542	−2.037	0.953
AIC	2465.421			2144.648			2119.452			942.834		
BIC	2509.133			2192.334			2167.138			990.520		

Table S4. Direct effects to predict depression with greyness at 250 m with four distributions (Poisson, Negative binomial, Gamma and Normal (log of depression)) (n = 393).

	Poisson Distribution			Negative Binomial Distribution			Gamma Distribution			Normal Distribution		
	Coefficient	95%	CI	Coefficient	95%	CI	Coefficient	95%	CI	Coefficient	95%	CI
Adverse Childhood Experiences	0.095	0.077	0.114	0.087	0.053	0.120	0.083	0.048	0.118	0.082	0.042	0.121
Social support index	−0.022	−0.030	−0.014	−0.024	−0.039	−0.009	−0.025	−0.041	−0.008	−0.034	−0.051	−0.017
Attachment to peers index	0.037	0.028	0.046	0.038	0.021	0.054	0.038	0.020	0.055	0.034	0.015	0.053
Healthy−food index	−0.010	−0.064	0.043	−0.015	−0.109	0.080	−0.015	−0.113	0.083	−0.006	−0.111	0.099
Physical−index activity	−0.084	−0.125	−0.042	−0.066	−0.135	0.003	−0.062	−0.132	0.007	−0.094	−0.173	−0.015
Body mass index	0.008	0.000	0.015	0.009	−0.004	0.022	0.009	−0.005	0.023	0.012	−0.002	0.027
Household income	−0.008	−0.015	0.000	−0.008	−0.020	0.004	−0.008	−0.020	0.004	−0.014	−0.028	−0.001
Age	0.267	0.160	0.374	0.289	0.113	0.464	0.297	0.121	0.473	0.312	0.120	0.505
Gender	−0.004	−0.021	0.013	−0.010	−0.040	0.019	−0.013	−0.044	0.017	−0.009	−0.042	0.024
Greyness at 250 m	0.410	0.184	0.636	0.394	0.009	0.779	0.379	−0.015	0.774	0.494	0.064	0.923
AIC	2461.012			2146.044			2120.929			941.146		
BIC	2504.752			2193.760			2168.645			988.863		

Table S5. Direct effects to predict depression with greyness at 500 m with four distributions (Poisson, Negative binomial, Gamma and Normal (log of depression)) (*n* = 393).

	**Poisson Distribution**			**Negative Binomial Distribution**			**Gamma Distribution**			**Normal Distribution**		
	**Coefficient**	**95%**	**CI**	**Coefficient**	**95%**	**CI**	**Coefficient**	95%	CI	Coefficient	95%	CI
Adverse Childhood Experiences	0.093	0.075	0.112	0.086	0.052	0.119	0.082	0.046	0.117	0.081	0.041	0.121
Social support index	−0.022	−0.030	−0.014	−0.025	−0.040	−0.009	−0.026	−0.042	−0.009	−0.035	−0.052	−0.017
Attachment to peers index	0.036	0.027	0.045	0.038	0.021	0.054	0.037	0.020	0.055	0.034	0.015	0.053
Healthy−food index	−0.016	−0.070	0.038	−0.015	−0.110	0.080	−0.015	−0.113	0.084	−0.006	−0.111	0.100
Physical−index activity	−0.084	−0.126	−0.043	−0.066	−0.136	0.003	−0.063	−0.133	0.007	−0.093	−0.172	−0.014
Body mass index	0.007	0.000	0.015	0.009	−0.004	0.022	0.009	−0.005	0.023	0.012	−0.002	0.027
Household income	−0.006	−0.023	0.011	−0.010	−0.040	0.019	−0.013	−0.044	0.017	−0.009	−0.043	0.024
Age	−0.008	−0.015	−0.001	−0.008	−0.020	0.004	−0.008	−0.020	0.004	−0.014	−0.028	−0.001
Gender	0.266	0.159	0.373	0.291	0.115	0.467	0.297	0.121	0.473	0.314	0.121	0.507
Greyness at 500 m	0.398	0.171	0.626	0.398	0.007	0.789	0.379	−0.022	0.781	0.485	0.046	0.924
AIC	2442.714			2136.72			2111.55			938.7326		
BIC	2486.341			2184.375			2159.205			986.3878		

Table S6. Direct effects to predict depression with greyness at 1000 m with four distributions (Poisson, Negative binomial, Gamma and Normal (log of depression)) (n = 394).

	**Poisson Distribution**			**Negative Binomial Distribution**			**Gamma Distribution**			**Normal Distribution**		
	Coefficient	95%	CI	Coefficient	95%	CI	Coefficient	95%	CI	Coefficient	95%	CI
Adverse Childhood Experiences	0.095	0.076	0.113	0.086	0.053	0.119	0.082	0.047	0.117	0.082	0.042	0.121
Social support index	−0.022	−0.030	−0.014	−0.024	−0.040	−0.009	−0.025	−0.042	−0.009	−0.035	−0.052	−0.017
Attachment to peers index	0.037	0.028	0.046	0.038	0.021	0.054	0.038	0.020	0.055	0.034	0.015	0.053
Healthy−food index	−0.010	−0.064	0.044	−0.015	−0.110	0.079	−0.015	−0.113	0.083	−0.007	−0.112	0.098
Physical−index activity	−0.085	−0.126	−0.043	−0.066	−0.136	0.003	−0.063	−0.132	0.007	−0.094	−0.173	−0.015
Body mass index	0.007	0.000	0.015	0.009	−0.005	0.022	0.009	−0.006	0.023	0.012	−0.003	0.027
Household income	−0.008	−0.015	−0.001	−0.008	−0.020	0.004	−0.008	−0.020	0.004	−0.015	−0.028	−0.001
Age	0.265	0.158	0.372	0.287	0.111	0.463	0.294	0.118	0.470	0.309	0.117	0.502
Gender	−0.004	−0.021	0.013	−0.009	−0.039	0.020	−0.012	−0.042	0.018	−0.009	−0.042	0.024
Greyness at 1000 m	0.362	0.141	0.582	0.345	−0.037	0.727	0.328	−0.067	0.722	0.419	−0.012	0.850
AIC	2463.552			2146.918			2121.783			942.5754		
BIC	2507.292			2194.634			2169.499			990.2916		

Table S7. Direct effects to predict depression with brownness at 250 m with four distributions (Poisson, Negative binomial, Gamma and Normal (log of depression)) (n = 393).

	**Poisson distribution**			**Negative binomial distribution**			**Gamma distribution**			**Normal distribution**		
	Coefficient	95%	CI	Coefficient	95%	CI	Coefficient	95%	CI	Coefficient	95%	CI
Adverse Childhood Experiences	0.10	0.08	0.11	0.09	0.05	0.12	0.08	0.05	0.12	0.08	0.04	0.12
Social support index	−0.02	−0.03	−0.01	−0.02	−0.04	−0.01	−0.02	−0.04	−0.01	−0.03	−0.05	−0.02
Attachment to peers index	0.04	0.03	0.05	0.04	0.02	0.05	0.04	0.02	0.06	0.03	0.02	0.05
Healthy−food index	−0.01	−0.07	0.04	−0.02	−0.11	0.08	−0.02	−0.12	0.08	−0.01	−0.12	0.09
Physical−index activity	−0.08	−0.12	−0.04	−0.07	−0.13	0.00	−0.06	−0.13	0.01	−0.09	−0.17	−0.01
Body mass index	0.01	0.00	0.01	0.01	0.00	0.02	0.01	−0.01	0.02	0.01	0.00	0.03
Household income	0.00	−0.02	0.01	−0.01	−0.04	0.02	−0.01	−0.04	0.02	−0.01	−0.04	0.02
Age	−0.01	−0.01	0.00	−0.01	−0.02	0.00	−0.01	−0.02	0.00	−0.01	−0.03	0.00
Gender	0.27	0.17	0.38	0.29	0.12	0.47	0.30	0.12	0.48	0.32	0.13	0.51
Brownness at 250 m	−0.37	−0.60	−0.14	−0.36	−0.75	0.04	−0.34	−0.75	0.06	−0.43	−0.87	0.00
AIC	2456.603			2141.956			2117.106			939.5244		
BIC	2500.315			2189.642			2164.792			987.2101		

Table S8. Direct effects to predict depression with brownness at 500 m with four distributions (Poisson, Negative binomial, Gamma and Normal (log of depression)) (*n* = 393).

	Poisson Distribution			Negative Binomial Distribution			Gamma Distribution			Normal Distribution		
	Coefficient	95%	CI	Coefficient	95%	CI	Coefficient	95%	CI	Coefficient	95%	CI
Adverse Childhood Experiences	0.094	0.075	0.112	0.086	0.053	0.120	0.082	0.047	0.118	0.082	0.042	0.122
Social support index	−0.022	−0.030	−0.014	−0.025	−0.040	−0.009	−0.026	−0.042	−0.009	−0.035	−0.052	−0.017
Attachment to peers index	0.036	0.027	0.045	0.038	0.021	0.054	0.037	0.020	0.055	0.034	0.015	0.053
Healthy−food index	−0.015	−0.069	0.040	−0.018	−0.113	0.077	−0.019	−0.117	0.080	−0.010	−0.116	0.096
Physical−index activity	−0.085	−0.126	−0.043	−0.066	−0.135	0.004	−0.062	−0.132	0.008	−0.092	−0.172	−0.013
Body mass index	0.007	0.000	0.014	0.008	−0.005	0.022	0.009	−0.006	0.023	0.012	−0.003	0.027
Household income	−0.006	−0.023	0.012	−0.010	−0.040	0.020	−0.013	−0.043	0.018	−0.008	−0.042	0.025
Age	−0.007	−0.015	0.000	−0.008	−0.020	0.004	−0.008	−0.020	0.004	−0.015	−0.028	−0.001
Gender	0.267	0.160	0.374	0.295	0.119	0.471	0.300	0.124	0.477	0.321	0.127	0.514
Brownness at 250 m	−0.423	−0.660	−0.185	−0.374	−0.775	0.027	−0.361	−0.774	0.052	−0.436	−0.883	0.010
AIC	2442.273			2132.424			2107.4			936.9033		
BIC	2485.901			2180.049			2155.084			984.5278		

Table S9. Direct effects to predict depression with brownness at 1000 m with four distributions (Poisson, Negative binomial, Gamma and Normal (log of depression)) (n = 393).

	Poisson Distribution			Negative Binomial Distribution			Gamma Distribution			Normal Distribution		
	Coefficient	95%	CI	Coefficient	95%	CI	Coefficient	95%	CI	Coefficient	95%	CI
Adverse Childhood Experiences	0.10	0.08	0.11	0.09	0.05	0.12	0.08	0.05	0.12	0.08	0.04	0.12
Social support index	−0.02	−0.03	−0.01	−0.02	−0.04	−0.01	−0.03	−0.04	−0.01	−0.03	−0.05	−0.02
Attachment to peers index	0.04	0.03	0.05	0.04	0.02	0.05	0.04	0.02	0.05	0.03	0.01	0.05
Healthy−food index	−0.01	−0.07	0.04	−0.02	−0.11	0.08	−0.02	−0.12	0.08	−0.01	−0.12	0.09
Physical−index activity	−0.08	−0.13	−0.04	−0.07	−0.14	0.00	−0.06	−0.13	0.01	−0.09	−0.17	−0.01
Body mass index	0.01	0.00	0.01	0.01	−0.01	0.02	0.01	−0.01	0.02	0.01	0.00	0.03
Household income	0.00	−0.02	0.01	−0.01	−0.04	0.02	−0.01	−0.04	0.02	−0.01	−0.04	0.02
Age	−0.01	−0.02	0.00	−0.01	−0.02	0.00	−0.01	−0.02	0.00	−0.01	−0.03	0.00
Gender	0.27	0.16	0.38	0.29	0.12	0.47	0.30	0.12	0.47	0.32	0.12	0.51
Brownness at 1000 m	−0.34	−0.56	−0.11	−0.32	−0.72	0.07	−0.31	−0.72	0.09	−0.38	−0.82	0.06
AIC	2458.436			2142.508			2117.586			940.4785		
BIC	2502.148			2190.193			2165.272			988.1642		

Table S10. Variance inflation factor to test for collinearity among variables and correlations.

Variance inflation factor

Variables in the models	VIF	1/VIF
Greenness at 250 m	1.03	0.966211
Adverse Childhood Experiences	1.10	0.863761
Social support index	1.22	0.818791
Attachment to peers index	1.15	0.870439
Healthy−food index	1.09	0.920600
Physical−index activity	1.08	0.922131
Body mass index	1.08	0.921872
Age	1.16	0.863761
Gender	1.04	0.964012
Income	1.05	0.954358
Mean VIF	1.10	

Table S11. Correlations among variables when considering greenness at 250 m.

	Depression	Greenness at 250 m	Adverse Childhood Experiences	Social support index	Attachment to peers index	Healthy−food index	Physical−index activity	Body mass index	Age	Gender	Income
Depression	1										
Greenness at 250 m	−0.0528	1									
Adverse Childhood Experiences	0.3198 *	−0.0591	1								
Social support index	−0.2464 *	−0.0078	−0.1759 *	1							
Attachment to peers index	0.2994 *	−0.0141	0.0935	−0.3111 *	1						
Healthy−food index	−0.0041	−0.0648	0.023	0.0919	0	1					
Physical−index activity	−0.0559	−0.0566	−0.0137	0.0698	−0.016	0.2183 *	1				
Body mass index	0.0788	0.0888	0.0893	−0.0031	−0.0021	0.0019	−0.1228	1			
Age	−0.0101	−0.0169	0.1213	−0.1882*	−0.0639	0.1235	−0.1346*	0.2284*	1		
Gender	0.0989	−0.0857	0.0589	0.1189	−0.0212	0.0413	−0.0809	−0.0199	0.018	1	

Income	−0.0853	−0.053	−0.1796*	0.0599	−0.0562	0.0012	−0.0228	0.0083	−0.0564	−0.0448	1

Table S12. Correlations among variables when considering greenness at 500 m.

	**Depression**	**Greenness at 500 m**	**Adverse Childhood Experiences**	**Social support index**	**Attachment to peers index**	**Healthy−food index**	**Physical−index activity**	**Body mass index**	**Age**	**Gender**	**Income**
Depression	1										
Greenness at 500 m	−0.0407	1									
Adverse Childhood Experiences	0.3198 *	−0.0683	1								
Social support index	−0.2464 *	−0.0323	−0.1759 *	1							
Attachment to peers index	0.2994 *	0.0119	0.0935	−0.3111 *	1						
Healthy−food index	−0.0041	−0.0659	0.023	0.0919	0	1					
Physical−index activity	−0.0559	−0.0513	−0.0137	0.0698	−0.016	0.2183*	1				
Body mass index	0.0788	0.0941	0.0893	−0.0031	−0.0021	0.0019	−0.1228	1			
Age	−0.0101	0.0003	0.1213	−0.1882 *	−0.0639	0.1235	−0.1346 *	0.2284 *	1		
Gender	0.0989	−0.067	0.0589	0.1189	−0.0212	0.0413	−0.0809	−0.0199	0.018	1	
Income	−0.0853	−0.0874	−0.1796 *	0.0599	−0.0562	0.0012	−0.0228	0.0083	−0.0564	−0.0448	1

Table S13. Correlations among variables when considering greenness at 1000 m.

	Depression	Greenness at 1000 m	Adverse Childhood Experiences	Social support index	Attachment to peers index	Healthy−food index	Physical−index activity	Body mass index	Age	Gender	Income
Depression	1										
Greenness at 1000 m	−0.0391	1									
Adverse Childhood Experiences	0.3198 *	−0.0578	1								
Social support index	−0.2464 *	−0.0188	−0.1759 *	1							
Attachment to peers index	0.2994 *	−0.0015	0.0935	−0.3111 *	1						
Healthy−food index	−0.0041	−0.0534	0.023	0.0919	0	1					
Physical−index activity	−0.0559	−0.0461	−0.0137	0.0698	−0.016	0.2183 *	1				
Body mass index	0.0788	0.0781	0.0893	−0.0031	−0.0021	0.0019	−0.1228	1			
Age	−0.0101	0.0035	0.1213	−0.1882 *	−0.0639	0.1235	−0.1346 *	0.2284 *	1		
Gender	0.0989	−0.0876	0.0589	0.1189	−0.0212	0.0413	−0.0809	−0.0199	0.018	1	
Income	−0.0853	−0.0886	−0.1796 *	0.0599	−0.0562	0.0012	−0.0228	0.0083	−0.0564	−0.0448	1

Table S14. Correlations among variables when considering greyness at 250 m.

	**Depression**	**Grayness at 250 m**	**Adverse Childhood Experiences**	**Social support index**	**Attachment to peers index**	**Healthy−food index**	**Physical−index activity**	**Body mass index**	**Age**	**Gender**	**Income**
Depression	1										
Greyness at 250 m	0.0887	1									
Adverse Childhood Experiences	0.3198 *	0.0057	1								
Social support index	−0.2464 *	−0.0239	−0.1759 *	1							
Attachment to peers index	0.2994 *	0.1229	0.0935	−0.3111 *	1						
Healthy−food index	−0.0041	−0.0813	0.023	0.0919	0	1					
Physical−index activity	−0.0559	−0.0311	−0.0137	0.0698	−0.016	0.2183*	1				
Body mass index	0.0788	−0.0169	0.0893	−0.0031	−0.0021	0.0019	−0.1228	1			
Age	−0.0101	−0.0685	0.1213	−0.1882 *	−0.0639	0.1235	−0.1346 *	0.2284 *	1		
Gender	0.0989	−0.0566	0.0589	0.1189	−0.0212	0.0413	−0.0809	−0.0199	0.018	1	
Income	−0.0853	−0.0073	−0.1796 *	0.0599	−0.0562	0.0012	−0.0228	0.0083	−0.0564	−0.0448	1

Table S15. Correlations among variables when considering greyness at 500 m.

	Depression	Grayness at 500 m	Adverse Childhood Experiences	Social support index	Attachment to peers index	Healthy−food index	Physical−index activity	Body mass index	Age	Gender	Income
Depression	1										
Greyness at 500 m	0.0894	1									
Adverse Childhood Experiences	0.3198*	0.0228	1								
Social support index	−0.2464*	0.0039	−0.1759*	1							
Attachment to peers index	0.2994*	0.1283*	0.0935	−0.3111*	1						
Healthy−food index	−0.0041	−0.0773	0.023	0.0919	0	1					
Physical−index activity	−0.0559	−0.0369	−0.0137	0.0698	−0.016	0.2183*	1				
Body mass index	0.0788	−0.0042	0.0893	−0.0031	−0.0021	0.0019	−0.1228	1			
Age	−0.0101	−0.057	0.1213	−0.1882*	−0.0639	0.1235	−0.1346*	0.2284*	1		
Gender	0.0989	−0.0607	0.0589	0.1189	−0.0212	0.0413	−0.0809	−0.0199	0.018	1	
Income	−0.0853	0.0028	−0.1796*	0.0599	−0.0562	0.0012	−0.0228	0.0083	−0.0564	−0.0448	1

Table S16. Correlations among variables when considering greyness at 1000 m.

	**Depression**	**Grayness at 1000 m**	**Adverse Childhood Experiences**	**Social support index**	**Attachment to peers index**	**Healthy−food index**	**Physical−index activity**	**Body mass index**	**Age**	**Gender**	**Income**
Depression	1										
Greyness at 1000 m	0.0877	1									
Adverse Childhood Experiences	0.3198*	0.0252	1								
Social support index	−0.2464*	−0.0073	−0.1759*	1							
Attachment to peers index	0.2994*	0.1484*	0.0935	−0.3111*	1						
Healthy−food index	−0.0041	−0.0712	0.023	0.0919	0	1					
Physical−index activity	−0.0559	−0.0476	−0.0137	0.0698	−0.016	0.2183*	1				
Body mass index	0.0788	0.0079	0.0893	−0.0031	−0.0021	0.0019	−0.1228	1			
Age	−0.0101	−0.0364	0.1213	−0.1882*	−0.0639	0.1235	−0.1346*	0.2284*	1		
Gender	0.0989	−0.0276	0.0589	0.1189	−0.0212	0.0413	−0.0809	−0.0199	0.018	1	
Income	−0.0853	−0.0062	−0.1796*	0.0599	−0.0562	0.0012	−0.0228	0.0083	−0.0564	−0.0448	1

Table S17. Correlations among variables when considering brownness at 250 m.

	Depression	Brownness at 250 m	Adverse Childhood Experiences	Social support index	Attachment to peers index	Healthy−food index	Physical−index activity	Body mass index	Age	Gender	Income
Depression	1										
Brownness at 250 m	0.0887	1									
Adverse Childhood Experiences	0.3198*	0.0057	1								
Social support index	−0.2464*	−0.0239	−0.1759*	1							
Attachment to peers index	0.2994*	0.1229	0.0935	−0.3111*	1						
Healthy−food index	−0.0041	−0.0813	0.023	0.0919	0	1					
Physical−index activity	−0.0559	−0.0311	−0.0137	0.0698	−0.016	0.2183*	1				
Body mass index	0.0788	−0.0169	0.0893	−0.0031	−0.0021	0.0019	−0.1228	1			
Age	−0.0101	−0.0685	0.1213	−0.1882*	−0.0639	0.1235	−0.1346*	0.2284*	1		
Gender	0.0989	−0.0566	0.0589	0.1189	−0.0212	0.0413	−0.0809	−0.0199	0.018	1	
Income	−0.0853	−0.0073	−0.1796*	0.0599	−0.0562	0.0012	−0.0228	0.0083	−0.0564	−0.0448	1

Table S18. Correlations among variables when considering brownness at 500 m.

	Depression	Brownness at 500 m	Adverse Childhood Experiences	Social support index	Attachment to peers index	Healthy−food index	Physical−index activity	Body mass index	Age	Gender	Income
Depression	1										
Brownness at 500 m	0.0894	1									
Adverse Childhood Experiences	0.3198*	0.0228	1								
Social support index	−0.2464*	0.0039	−0.1759*	1							

Attachment to peers index	0.2994*	0.1283*	0.0935	−0.3111*	1						
Healthy−food index	−0.0041	−0.0773	0.023	0.0919	0	1					
Physical−index activity	−0.0559	−0.0369	−0.0137	0.0698	−0.016	0.2183*	1				
Body mass index	0.0788	−0.0042	0.0893	−0.0031	−0.0021	0.0019	−0.1228	1			
Age	−0.0101	−0.057	0.1213	−0.1882*	−0.0639	0.1235	−0.1346*	0.2284*	1		
Gender	0.0989	−0.0607	0.0589	0.1189	−0.0212	0.0413	−0.0809	−0.0199	0.018	1	
Income	−0.0853	0.0028	−0.1796*	0.0599	−0.0562	0.0012	−0.0228	0.0083	−0.0564	−0.0448	1

Table S19. Correlations among variables when considering brownness at 1000 m.

	**Depression**	**Brownness at 1000 m**	**Adverse Childhood Experiences**	**Social support index**	**Attachment to peers index**	**Healthy−food index**	**Physical−index activity**	**Body mass index**	**Age**	**Gender**	**Income**
Depression	1										
Brownness at 1000 m	0.0877	1									
Adverse Childhood Experiences	0.3198*	0.0252	1								
Social support index	−0.2464*	−0.0073	−0.1759*	1							
Attachment to peers index	0.2994*	0.1484*	0.0935	−0.3111*	1						
Healthy−food index	−0.0041	−0.0712	0.023	0.0919	0	1					
Physical−index activity	−0.0559	−0.0476	−0.0137	0.0698	−0.016	0.2183*	1				
Body mass index	0.0788	0.0079	0.0893	−0.0031	−0.0021	0.0019	−0.1228	1			
Age	−0.0101	−0.0364	0.1213	−0.1882*	−0.0639	0.1235	−0.1346*	0.2284*	1		
Gender	0.0989	−0.0276	0.0589	0.1189	−0.0212	0.0413	−0.0809	−0.0199	0.018	1	
Income	−0.0853	−0.0062	−0.1796*	0.0599	−0.0562	0.0012	−0.0228	0.0083	−0.0564	−0.0448	1

Table S20. Diagnostics Structural Equation Models.

	Greenness at 250 m	Greenness at 500 m	Greenness at 1000 m	Greyness at 250 m	Greyness at 500 m	Greyness at 1000 m	Brownness at 250 m	Brownness at 500 m	Brownness at 1000 m
SRMR	0.059	0.059	0.059	0.058	0.059	0.058	0.058	0.059	0.058
CD	0.157	0.155	0.155	0.175	0.175	0.179	0.174	0.176	0.181

STDR Standardized root mean squared residual; CD Coefficient of determination.

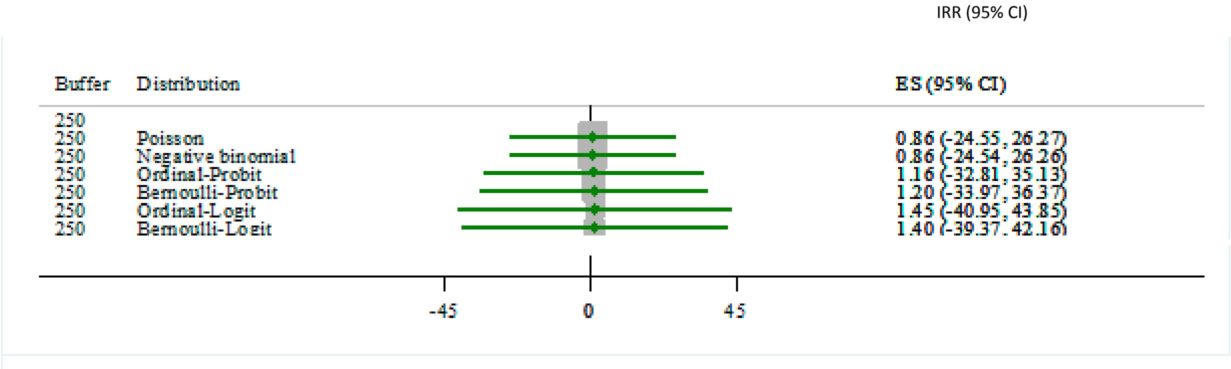

Figure S8. Social support as a pathway between greenness and depression (ordinal variable), in 250 m buffer.

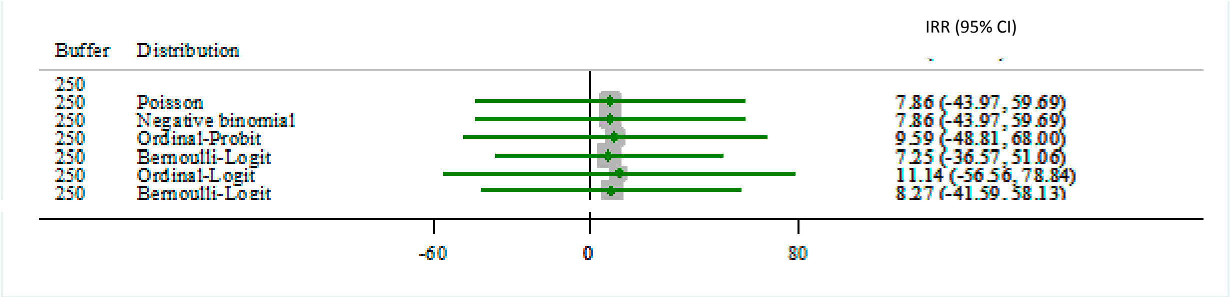

Figure S9. Peer alienation as a pathway between greenness and depression (ordinal variable), in 250 m buffer. Buffer indicates distances in meters around the geocoded address of residence of each study participant. Distribution indicates the distribution in which depression was modeled. When the normal distribution was assumed the values of depression were log transformed. IRR are incidence rate ratios. CI is confidence interval. All models were adjusted by Age, Sex, Healthy−food index, Physical−Activity index, Body mass index, Household income, Adverse Childhood Experiences, Social support index, and Peer alienation index.

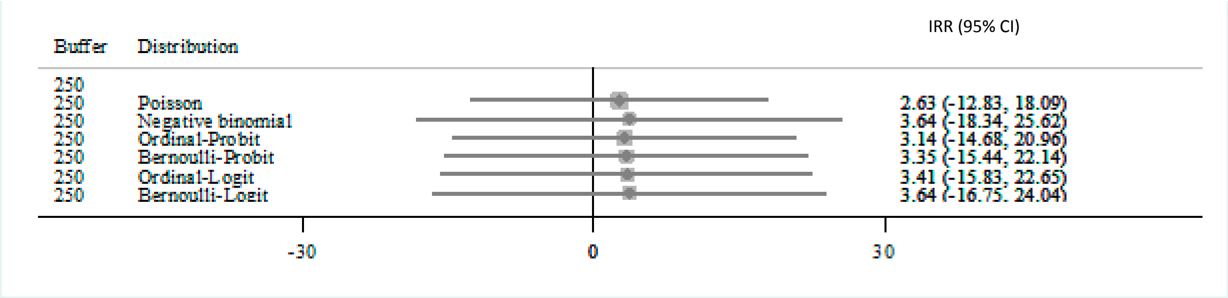

Figure S10. Social support as a pathway between greyness and depression (ordinal variable) in 250 m buffer.

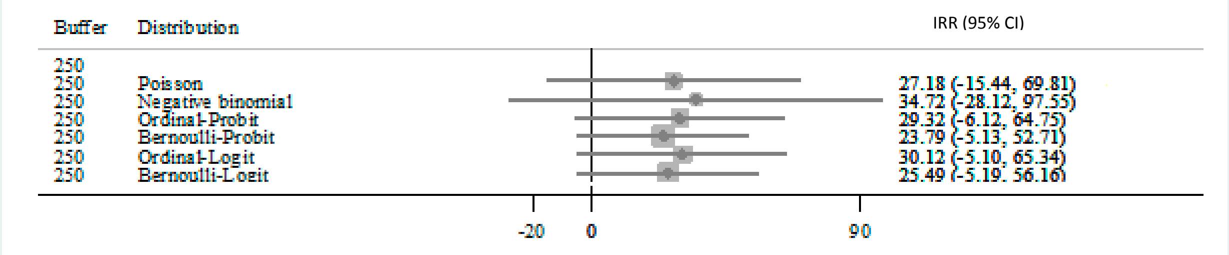

Figure S11. Peer alienation as a pathway between greyness and depression (ordinal variable) in 250 m buffer. Buffer indicates distances in meters around the geocoded address of residence of each study participant. Distribution indicates the distribution in which depression was modeled. When the normal distribution was assumed the values of depression were log transformed. IRR are incidence rate ratios. CI is confidence interval. All models were adjusted by Age, Sex, Healthy−food index, Physical−Activity index, Body mass index, Household income, Adverse Childhood Experiences, Social support index, and Peer alienation index.

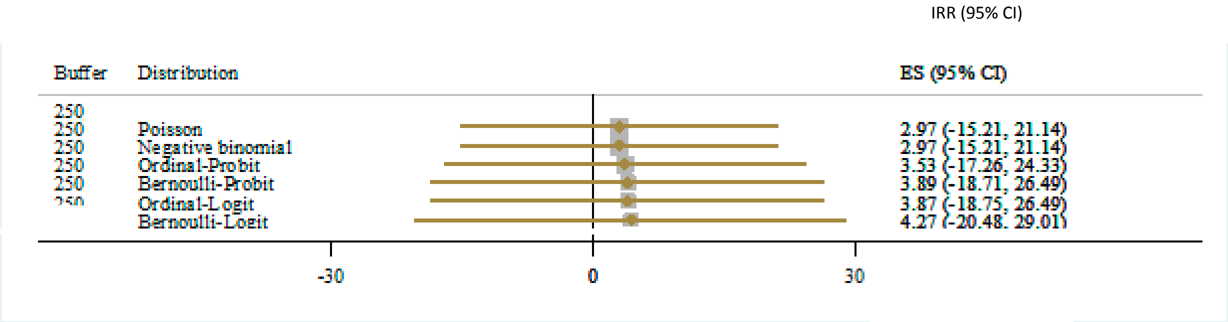

IRR (95% CI)

Figure S12. Social support as a pathway between brownness and depression ([illegible] 250 m buffer.

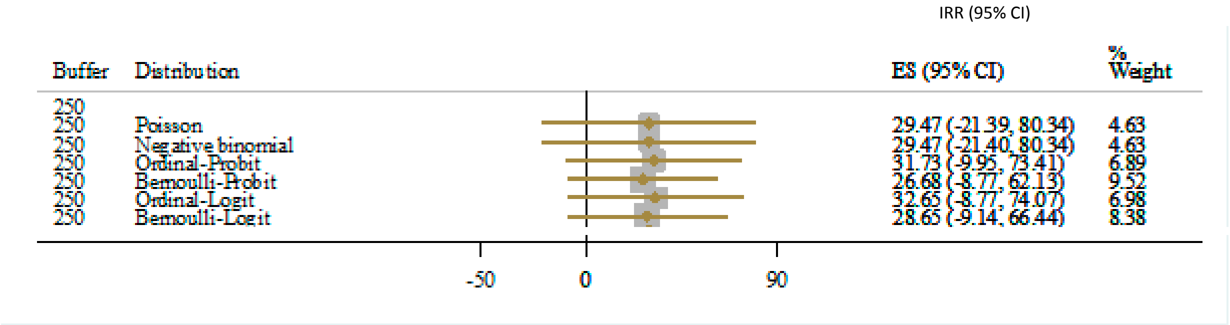

Figure S13. Peer alienation as a pathway between brownness and depression (as a) continuum and b) ordinal variable) in 250 m buffer. Buffer indicates distances in meters around the geocoded address of residence of each study participant. Distribution indicates the distribution in which depression was modeled. When the normal distribution was assumed the values of depression were log transformed. IRR are incidence rate ratios. CI is confidence interval. All models were adjusted by Age, Sex, Healthy−food index, Physical−Activity index, Body mass index, Household income, Adverse Childhood Experiences, Social support index, and Peer alienation index.
